# Macon Medical Society

**Published:** 1885-05

**Authors:** 


### MACON MEDICAL SOCIETY.

STATED MEETING, March 24, 1885.

The President, Dr. Stevens, in the chair.

Dr. P. H. Wright read an essay entitled :

### DRESSING OF NEW-BORN INFANTS.

This is a trite and old-time subject, but one which, although much talked of by old grannies and anxious mothers, doubtless, since the time of Mother Eve, has not, I fear, received that attention its importance demands from the profession. Medical men, and even our standard works on obstetrics, do not give the subject that attention its importance demands. We have too much left this very important part of our duties in the lying-in chamber in the hands of ignorant nurses, who too often think they know better how to dress and take care of the body than the physician.

As one has written, if there be one custom of time-honored folly, which we have continued to this day in the "lying-in chamber," it is that absurd and cruel system of the first dressing.

I shall endeavor to present to you to-night the usual routine which all of you have doubtless witnessed many times, and which will be a familiar scene to you.

So soon as the cord is tied and severed, the body is given to an employed nurse, some *wise* and *experienced* neighbor or friend.

"What will she do with it," may best be solved by watching her. First, she huddles it up in an old shawl or other garment. She is very careful to cover its head, so as scarcely to leave even a *chink* through which a *breath* of air can pass, as if it were a young puppy she would smother, or rid the world of an infant cat.

Hot water, lard, soap and towels have been prepared, with bundles of old linen or cotton rags, and a basket containing the clothing for the little stranger.

The good woman now turns to the blazing fire, that the body may not "take cold," and while the youngster implores with yells and cries, she bakes its tender skin on one side, while, having thoroughly greased it with lard from head to foot, she dobbles its head, eyes, mouth and body with soap-suds of frequently very bad soap. Having scrubbed and washed the child until she is satisfied that it is "*clean*," she then turns her attention to the cord, upon which (in the language of another) she frequently deposits, slyly, some pestiferous saliva—"*it's healin'*," she says—and now she follows authority. She cuts a hole in the centre of a piece of scorched cloth—old linen preferred—through which she draws the cord. She folds this carefully around the cord, it being well greased. And then, laying it smoothly up the child's belly, she then puts on a binder, usually drawing this so tight as effectually to stop all abdominal breathing. The diaper is now put on, usually large enough to almost envelop the entire body. Now she puts on a little garment called a shirt, which is in fact usually without body, neck or sleeves, so far as protection goes. Then a flannel skirt pinned tightly around the waist, then another waist skirt, usually stiffly starched, and then a dress, and now the baby is presentable.

The doctor sees it is all right and goes home. He hears not, within an hour or two, the stifled screams of compressed lungs and belly, that with every breath are expanding the chest and bulging the umbilicus ; and the nurse wisely says it is "colicy," for which it must be drenched with some decoction of catnip, brandy, paregoric, "soothing" syrup, etc. The next visit the doctor is told the baby is a good baby, and getting on all right, except only a little "colicy," which she can cure, and away the doctor goes where he can't hear the baby cry, and see its nose greased for the snuffles, and its stomach dosed for the "colick" on account, as to the snuffles, of its being exposed during the washing, and as to the "colick" of the cord having become a half putrid, half dying mass, glued and ulcerating to the tender belly. This picture may be overdrawn for some cases, but upon carefully watching you will find some of the above-named outrages, at least, in force.

In my own experience, I have found the following method gives the most comfort to all hands, by giving the baby no excuse for those cries, which are seldom heard, if the baby is not uncomfortable : 1st. As a rule, especially in cold weather, the baby should not be bathed for several hours—12, or better still, 24.

This rule I have sometimes found it hard to enforce, for the reason that the women are very particular that the babe should be washed clean as quickly as possible. But it is *important*, and should be insisted on, when we consider the great difference in the temperature of the mother's womb and the atmosphere outside, as well as the suddenness of the change, we can readily understand how easy it is for the young child to become chilled, thereby exposing it to disease.

In my opinion this practice of washing and dressing the babe so soon as it is born is one of the most fruitful sources of influenza, nasal catarrh, etc.

2d. The child should be warmly and comfortably dressed, with easy, loose fitting garments suspended from the shoulders (flannel next to the skin), being careful to allow no constriction at any part of the body. As to the dressing of the cord, I have for a long time been in the habit of simply enveloping the cord with raw, clean cotton, wrapping it snugly around the cord. This is usually all the dressing it will need, and in a few days the cord will come off without causing any discomfort.

The belly-band should only be put on just snugly enough to keep the cord secured from falling about, but not in the least to impede the free expansion of the abdominal walls.

If we should give this seemingly unimportant subject more of our personal attention, and see to it that so far as the first dressing and other management of the young child is concerned, it is properly done, we would save many babes from much discomfort, if not, as I believe, frequently from serious disease.

The proper management of the young infant is of *far greater* importance to secure it from future ills than medical men have, as a class, seemed to think.

Dr. C. H. Hall: I have listened with interest to Dr. Wright's paper, and agree with him in that much harm is done by bathing the child too soon after birth. My practice is to wrap the child in woolen cloth immediately, and put aside to attend to the mother. After she has been attended to, I have the child greased and placed bodily in water, and thus every part is covered, and there is no chance of exposure. I think it bad practice to hold the child upon the knee and dab with a wet cloth, for the child becomes chilled, and the foundation of the diseases of infancy is often laid by this carelessness. If the vitality of the child is not fully established, I think it advisable to wait, as Dr. Wright has said, at least ten or twelve hours. I never interfere with the cord until the child is bathed, and I use the ordinary dressing. All clothing should be loose, and the clothes next the skin should be soft and unstarched.

Another evil, I think, is too much physicing. Opium, I think, should never be used; the German camomile is *par excellance* the best remedy for quieting; it is a nervine, and at the same time I think it has a good effect on the bowels. Castor oil is frequently used, but it is, in my opinion, bad, as it causes a hemorrhoidal tendency by determining the blood to the anal region; I am in the habit of using recently the cascara cordial.

